# Dopamine Self-Polymerization as a Simple and Powerful Tool to Modulate the Viscoelastic Mechanical Properties of Peptide-Based Gels

**DOI:** 10.3390/molecules26051363

**Published:** 2021-03-04

**Authors:** Galit Fichman, Joel P. Schneider

**Affiliations:** Chemical Biology Laboratory, National Cancer Institute, National Institutes of Health, Frederick, MD 21702, USA; gali.fichman@nih.gov

**Keywords:** dopamine, peptide, self-assembly, hydrogel

## Abstract

Dopamine is a small versatile molecule used for various biotechnological and biomedical applications. This neurotransmitter, in addition to its biological role, can undergo oxidative self-polymerization to yield polydopamine, a robust universal coating material. Herein, we harness dopamine self-polymerization to modulate the viscoelastic mechanical properties of peptide-based gels, expanding their ever-growing application potential. By combining rapid peptide assembly with slower dopamine auto-polymerization, a double network gel is formed, where the fibrillar peptide gel network serves as a scaffold for polydopamine deposition, allowing polydopamine to interpenetrate the gel network as well as establishing crosslinks within the matrix. We have shown that triggering the assembly of a lysine-rich peptide gelator in the presence of dopamine can increase the mechanical rigidity of the resultant gel by a factor of 90 in some cases, while retaining the gel’s shear thin-recovery behavior. We further investigate how factors such as polymerization time, dopamine concentration and peptide concentration alter the mechanical properties of the resultant gel. The hybrid peptide–dopamine gel systems were characterized using rheological measurements, circular dichroism spectroscopy and transmission electron microscopy. Overall, triggering peptide gelation in the presence of dopamine represents a simple yet powerful approach to modulate the viscoelastic mechanical properties of peptide-based gels.

## 1. Introduction

Dopamine, a catecholamine neurotransmitter, has emerged in the last decade as a versatile building block for countless biotechnological and biomedical applications [1,2,3]. As first reported by Messersmith and coworkers [4], oxidative self-polymerization of dopamine in an aqueous alkaline solution [5] can be used to yield polydopamine film, a robust universal coating material. The low cost of dopamine and the simplicity of polydopamine deposition on virtually any object, advanced polydopamine research and usage, though the exact self-polymerization mechanism as well as polydopamine structure are still in debate [1,2,3]. Nevertheless, studies on polydopamine films deposited onto surfaces under various conditions, revealed the importance of factors such as solution pH, dopamine concentration and self-polymerization time in controlling film thickness [2]. The polydopamine film exhibits versatile chemistry, capable of forming covalent crosslinks with several functional groups including thiols and amines, and physical interactions such as hydrogen-bonds and cation-π interactions [1,6]. Indeed, polydopamine film can be exploited as either a primary coating agent without further modification or as a primer for a secondary coating. Furthermore, in addition to surface functionalization, Adams and coworkers have shown that dopamine auto-oxidation can be used to trigger gelation, where the resultant gels can display antibacterial activity [7]. We became interested in exploring the possibility that polydopamine could be used to increase the mechanical rigidity of peptide-based gels by acting as a crosslinker and interpenetrant.

Peptide-based supramolecular gels, by themselves, represent an important class of viscoelastic material suitable for various applications [8,9]. Advantages of using self-assembling peptides as building blocks to form hydrogels include facile peptide synthesis and ease of peptide functionalization with desired chemical moieties or biological recognition motifs. Indeed, owing to their unique chemical, biological and physical properties, peptide-gel scaffolds have been used for drug delivery, wound healing, cell culturing and tissue engineering, where the gels’ nanofibrous structure and stiffness serve as an extracellular matrix (ECM) mimetic. The viscoelastic mechanical behavior of peptide gels often define how they are used [10,11]. Thus, the ability to control and fine-tune the mechanical properties of a gel is highly desired. For instance, by altering the stiffness of peptide-gels, in vitro disease-relevant breast cancer models were developed, where gel stiffness was matched to either normal breast (<1 kPa) or breast tumor (>1 kPa) tissue [12]. Peptide gel rigidity can also be modulated to direct the fate of stem cells [11,13,14]. For example, seeding human mesenchymal stem cells (hMSCs) on soft peptide-gels led to maximal spreading and elongation of the cells, with higher expression of βIII-tubulin neuronal marker, indicating neuronal differentiation [14]. Indeed, considerable effort has been made to modulate and enhance the mechanical rigidity of peptide-based gels, in order to further expand their potential use [15]. In general, one can increase their rigidity by using higher peptide concentrations for self-assembly, changing solution conditions or altering peptide sequence [16,17,18,19,20]. Further, efforts are still made to introduce new, broader approaches to modulate the rigidity of peptide-gels, and this remains an active area of exploration. More recent efforts include the installation of crosslinks into the supramolecular gel network using physical, enzymatic, or chemical crosslinking mechanisms [21,22,23,24,25,26,27,28,29], and the design of hybrid polymer/peptide systems, where a polymeric component is introduced into a peptide-gel system [30,31,32,33].

**Figure 1:**
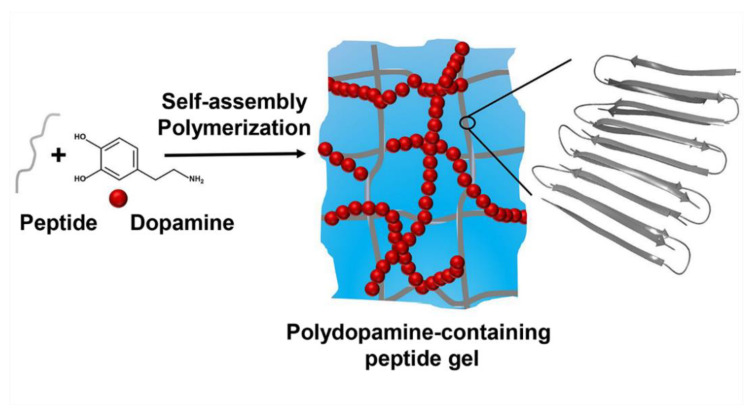
Conceptual scheme describing the assembly and gelation of peptide gelator in the presence of dopamine. Occurrence of rapid peptide assembly along with a slower dopamine auto-polymerization reaction results in a gel with increased mechanical rigidity.

We envisioned that dopamine auto-polymerization could be used to form an interpenetrating network that could crosslink a peptide network via reacting with the lysine residues of assembled peptide, forming a double network hydrogel, Figure 1. Thus, polydopamine should increase the cohesion of the system, enhancing the mechanical rigidity of the gel matrix. Herein, we show that when peptide self-assembly is initiated in the presence of dopamine, a dramatic increase in storage modulus is realized.

We had previously developed a class of amphiphilic peptides that can undergo triggered self-assembly into supramolecular hydrogels [34]. The parent MAX1 hydrogelator is a 20-residue peptide composed of two β-strands of alternating valine and lysine residues, connected by a type II′ β turn motif structure (V^D^PPT). MAX1 assembly and gelation were well-characterized over several length scales, using rheology and various microscopic and spectroscopic techniques, including cryo-TEM, AFM and solid-state NMR [35,36,37,38,39]. In water and under acidic conditions MAX1 is unstructured due to repulsive interactions between its protonated lysine residues. Yet, peptide assembly is triggered in the presence of saline buffer that increases the solution pH and ionic strength, effectively reducing the inter-lysine electrostatic interactions. The peptide rapidly assembles, adopting an amphiphilic β-hairpin conformation in its fibrillar, self-assembled state. Resultant fibrils are monomorphic, characterized by a width and a height of approximately 3 and 2 nm, respectively. Each fibril is composed of a bi-layered cross β-structure [39]. The resulting network formed by these fibrils gives rise to a self-supporting gel.

The rapid formation of fibrillar network that percolates the entire sample volume (<1 min. at 1 wt.% MAX1 peptide) [40], coupled with the slower self-polymerization process of dopamine, should allow the establishment of a well-defined peptide-based fibrillar template for polydopamine deposition. As polydopamine is forming, it can then interpenetrate the peptide gel network as well as act as a crosslinking agent by reacting with some of the lysine residues that are heavily displayed on the solvent exposed surface of the fibrils, introducing covalent crosslinks within the supramolecular network [1]. Furthermore, lysine residues can participate in non-covalent cation–π interactions [6], further rigidifying the gel network.

Despite the extensive studies on dopamine self-polymerization, the exact polymerization process is not fully understood, and several models were proposed to describe the mechanism, ranging from covalent coupling to non-covalent self-assembly of subunits [1,2,41]. Yet, in all theories of polydopamine formation the initial driving force for dopamine self-polymerization is dopamine oxidation at alkaline pH by dissolved oxygen, where the catechol moiety of polydopamine oxidizes to quinone. Several factors can further affect the self-polymerization process including, among others [42], dopamine concentration and self-polymerization time, factors investigated in this report.

## 2. Results and Discussion

We first examined the hybrid gel obtained by the gelation of 1 wt.% MAX1 peptide in the presence of 10 mM dopamine at pH 7.4. A concentration of 10 mM dopamine was chosen as it is known that dopamine spontaneously self-polymerizes in the presence of molecular oxygen under alkaline conditions (pH > 7.5) at a concentration ≥ 2 mg/mL (~10 mM) [3]. To introduce polydopamine into the MAX1 gel system, MAX1 and monomeric dopamine were initially mixed together in cold water, where MAX1 is also monomeric and unfolded, and auto-polymerization of dopamine is not favorable [3]. HEPES buffer (pH 7.4) was introduced to the mixture solution to trigger MAX1 gelation and promote dopamine auto-polymerization. To further facilitate MAX1 gelation and dopamine auto-polymerization, the temperature was adjusted to 37 °C. Increasing the temperature can induce the hydrophobic effect to promote MAX1 assembly [43,44], as well as accelerate polydopamine formation [45]. As a control, the MAX1 gel was prepared similarly, in the absence of dopamine.

**Figure 2:**
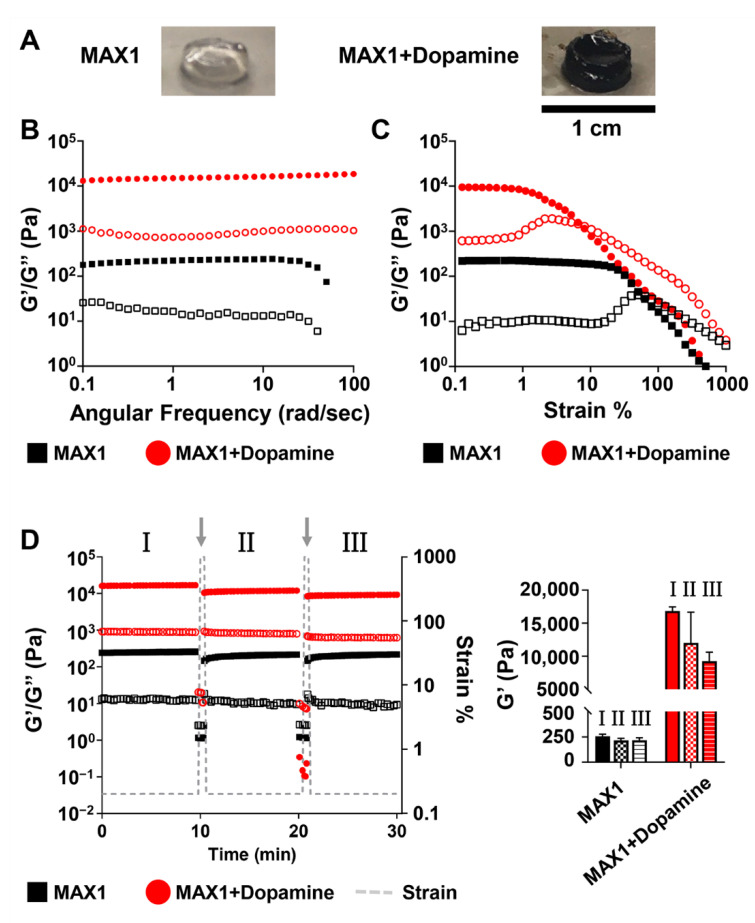
Rheological studies of the 1 wt.% MAX1-dopamine gel system. (**A**) The corresponding images of 1 wt.% MAX1 gels 3 days post triggering gelation with or without 10 mM dopamine (**B**) Frequency and (**C**) Strain sweep rheological data collected at 0.2% strain and 6 rad/s frequency, respectively, for pre-formed gels. Closed and open symbols represent storage (G′) and loss (G″) modulus, respectively. (**D**) Recovery of gels was followed after two repetitive shear-thinning cycles. In each cycle, the G′ of the gels was initially measured at low strain for 10 min (0.2% strain), followed by 30 s measurements under high strain (1000% strain) that ruptures the gel network. Then, gel recovery is determined by measuring the G′ again at low strain (0.2% strain) for 10 min. All measurements were performed at a frequency of 6 rad/s. Measurement segments performed within the LVE regime (0.2% strain, 6 rad/s frequency) are marked as I, II, III. Grey arrows indicate the 30 s measurement segments performed under high strain (1000%). To the right are bar graphs of the G′ values collected for the gels at the end of each I, II and III segments. All rheological data represent the average G′ and G″ obtained from at least three independent measurements.

As seen in Figure 2A, three days post triggering the gelation of MAX1 in the presence of dopamine, a homogeneous dark brown gel was obtained, in contrast to the colorless gel formed by MAX1 alone. The dark color of the gel serves as indication of polydopamine formation within the gel matrix, as was reported in other polydopamine systems [46,47]. Rheological studies were performed on the MAX1 and MAX1-polydopamine hybrid gel systems. In these studies, gels were formed in molds and transferred to the rheometer for subsequent analysis. Dynamic frequency sweep and strain sweep experiments were performed at 0.2% strain and 6 rad/s frequency, respectively, within the viscoelastic linear (LVE) regime (Figure 2B,C). The frequency sweep shows a significant 66-fold increase in the mechanical rigidity of the MAX1-polydopamine gel in comparison to the MAX1 gel (16,852 ± 613 vs. 257 ± 20 Pa). In both gel systems the G′ values were about an order of magnitude larger than the G″ which is a characteristic behavior of a viscoelastic gel. The strain sweep shows that the hybrid gel is more brittle than MAX1, starting to yield and display fluid-like behavior (G′ < G″) at lower strain%. The brittleness of the hybrid gel suggests that covalent crosslinks were successfully installed into the physical peptide network.

To study the shear-thin/recovery properties of the gels, shear-thinning cycles were applied as outlined in Figure 2D. Here, following a ten-minute time sweep within the LVE regime (0.2% strain, 6 rad/s), high strain (1000% strain) was applied to shear-thin each gel (Figure 2D, grey arrow). The strain is then decreased back to 0.2% and the G′ and G″ values of the gels are recorded again within the LVE regime for ten minutes. Upon performing two such shear cycles we observed that the MAX1-polydopamine gel displays shear-thin/recovery properties as did the MAX1 gel, which has previously been shown to rapidly restore its stiffness as a function of time after being deformed by 1000% strain [48]. The G′ values of the recovering MAX1 gels after recovery were 215 ± 20 and 217 ± 24 Pa, following the first and the second shear thin cycles, respectively. The recovery behavior of the MAX1 gels, expressed in percent recovery with respect to the initial pre-sheared G′ value, was similar in both the first and second shearing cycles, displaying about 84% recovery. In contrast, the G′ values of the recovered hybrid MAX1-dopamine gel differ between the two shear cycles (12,020 ± 4638 and 9257 ± 1371 Pa, corresponding to 71 ± 25 and 55 ± 8% recovery). The decrease in G′ values as a function of the shearing cycle indicates that the hybrid MAX1-dopamine gel network formed under these conditions fails to restore all of its mechanical integrity upon shear-thinning. Nevertheless, the recovered G′ values of the hybrid MAX1-dopamine gels, even after two successive shear-thinning cycles, were still about 36 times higher than the G′ values of MAX1 gels prior to shearing.

**Figure 3:**
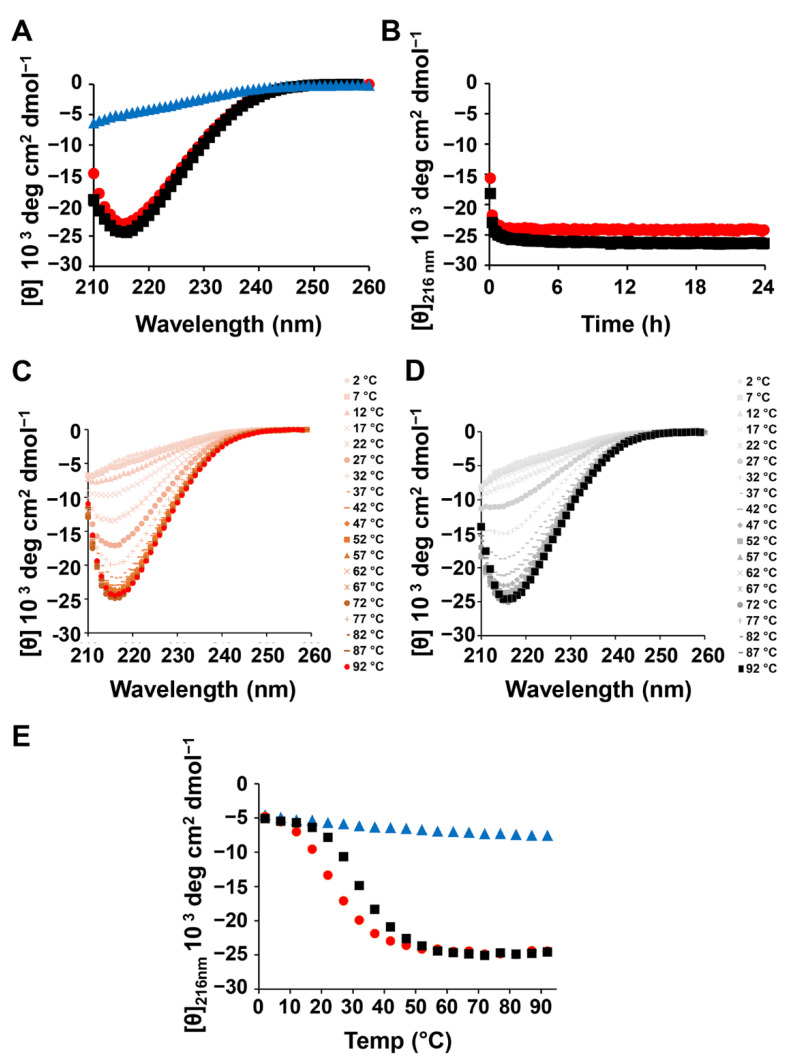
Secondary structural analysis of the MAX1-dopamine gel system using circular dichroism (CD) spectroscopy. (**A**) CD spectra at 37 °C were collected for 1 wt.% MAX1 in water (blue triangles) and in buffer, in the absence or presence of dopamine (black squares and red circles, respectively). Data collection was initiated after 20 min of sample equilibration at 37 °C. (**B**) Rate of β-sheet formation of MAX1 in the absence or presence of dopamine. The evolution of β-sheet is monitored by recording [θ]_216_ as a function of time for 1 wt.% peptide solution at 37 °C, starting 5 min after peptide assembly is initiated by the addition of buffer (pH 7.4). (**C**,**D**) Temperature-dependent wavelength CD spectra of 1 wt.% MAX1 in buffer, in the absence or presence of dopamine (**C**,**D**, respectively). (**E**) The evolution of β-sheet as a function of temperature is presented for MAX1 in water (blue triangles) and in buffer, in the absence or presence of dopamine (black squares and red circles, respectively).

In the absence of dopamine, MAX1 is known to self-assemble into β-sheet rich fibrils where the peptide is folded into an amphiphilic β-hairpin conformation. Circular dichroism (CD) spectra of both MAX1 and MAX1-dopamine were collected and compared to elucidate the influence of dopamine on MAX1 assembly and folding. As seen in Figure 3A, MAX1 in water is unstructured, displaying a characteristic random-coil CD spectrum. Yet, in saline buffer (pH 7.4), where peptide assembly and gelation are triggered, the peptide adopts β-sheet structure, regardless of the absence or presence of dopamine, showing a characteristic β-sheet spectrum with a minimum at 216 nm for both samples. To examine more closely the assembly kinetics of the peptide in the absence or presence of dopamine, the evolution of β-sheet formation was monitored by recording the mean residue ellipticity at 216 nm ([θ]_216_) as a function of time, immediately after gelation is initiated. Figure 3B shows that at 37 °C in the early stages of self-assembly β-sheet formation is fast, where promptly about 5 min after triggering assembly, the recorded [θ]_216_ values were about −18,000 and −16,000 deg cm^2^ dmol^−1^ for MAX1 and MAX1-dopamine gel systems, respectively. These values were found to be correlated with gel formation, where a previous study has demonstrated that the gel point of MAX1 corresponds to [θ]_216_ values between −10,000 and −12,000 deg cm^2^ dmol^−1^, representing the minimum amount of fibrillar β-sheet structure needed to form a percolated gel network [36]. For both samples a similar rate of β-sheet formation was observed, reaching a plateau region, which is indicative of the completion of β-sheet formation, after about 15 min. Furthermore, the β-sheet content remained unchanged up to 24 h, the last examined time point. Temperature dependent-CD spectra were also collected for MAX1 in the absence and presence of dopamine, Figure 3C,D, respectively. MAX1 is known to undergo thermally induced self-assembly. At low temperatures, the peptide is unfolded. Raising the temperature drives the hydrophobic effect, resulting in peptide assembly [40,43]. As summarized in Figure 3E similar transitions were observed for the two samples, where at low temperature, below 12 °C, the peptide was unstructured and peptide assembly started only at higher temperatures of about 22 or 17 °C, in the absence or presence of dopamine, respectively. In pure water, MAX1 did not displayed a β-sheet structural transition at all examined temperatures (Figure 3E, Figure S1, Supplementary Materials). CD data were also collected for dopamine alone in buffer, but no contribution to the CD signal at 216 nm was observed, neither at 37 °C as a function of time (Figure S2) nor at different temperatures (Figure S3). Collectively, the CD data of the MAX1-dopamine gel system suggest that under the examined conditions (1 wt.% MAX1, pH 7.4 37 °C) dopamine does not influence the ability of MAX1 to self-assemble and suggests that MAX1 quickly assembles, forming a network before dopamine auto-polymerizes to any great extent. As such, the formed peptide-gel network can serve as a scaffold for polydopamine deposition, where polydopamine can physically interpenetrate the fibrillar network as well as covalently crosslink to the fibers. Covalent crosslinks can be formed from lysine side chains reacting with quinone intermediates formed during dopamine auto-polymerization. If dopamine polymerization and MAX1 assembly occurred at a similar rate, one might expect peptide assembly to be disrupted by the formation of covalent bonds (premature crosslinks) between dopamine and monomeric peptide before it had a chance to assemble.

**Figure 4:**
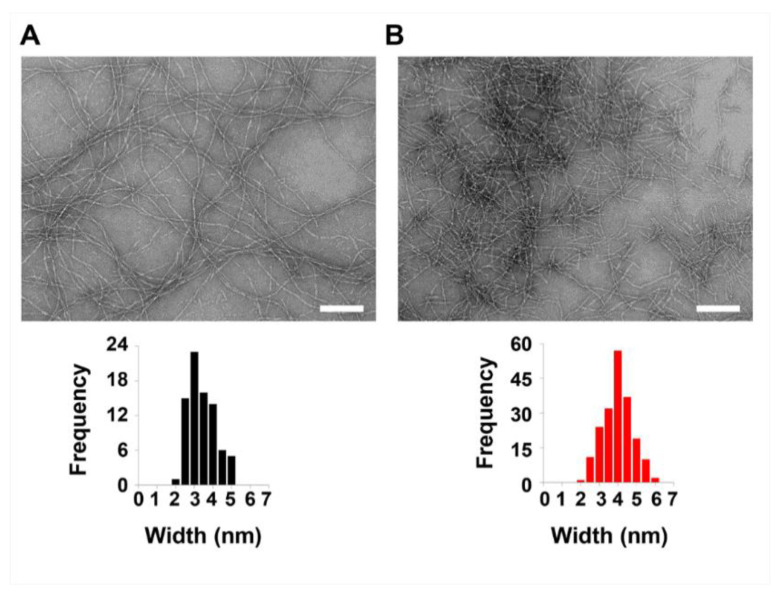
Representative TEM micrographs showing fibrils isolated from 1 wt.% fibrillar gel networks 3 days after gelation is triggered in the absence or presence of 10 mM Dopamine (**A** and **B**, respectively). Scale bar = 100 nm. Widths of individual fibrils of each sample were determined using ImageJ software, by measuring width of fibrils from 3 separate micrographs, representing different location of the fibrils on the grid, *n* = 193 and *n* = 80 for the gel with without the dopamine, respectively.

To explore the effect of polydopamine deposition on the morphology of MAX1 fibrils, transmission electron microscopy (TEM) was used. Here, three days post-triggering the assembly of MAX1 in the absence or presence of dopamine, the gel’s fibril network was diluted with water to allow visualization of distinct fibrils. In the absence of dopamine, monomorphic MAX1 fibrils are formed with a width of about 3 nm (Figure 4A, Figure S4). In contrast, dilution of the hybrid MAX1-dopamine gel system, resulted in clusters of entangled or laminated fibrils. Moreover, in comparison to the fibrils that constituted the MAX1 gel, the dopamine/peptide fibrils were of inconsistent length, typically shorter, and slightly wider, with a width of about 4 nm (Figure 4B and Figure S4). The subtle change to the width of the fibrils in the presence of dopamine may be indicative of polydopamine film formation on the fibrils. Although the TEM micrographs report only on the 2D local morphology of the fibrils and not the 3D network from which they were harvested, the observed differences in fibril morphology and the altered shear-thin/recovery properties of the gel when dopamine is included, is consistent with an altered 3D network, such as that produced by polydopamine deposition.

Polydopamine film formation via static self-polymerization (i.e., without external agitation), is a slow process, taking 24 h to form a film having a thickness of 50 nm [4]. The thickness of the polydopamine film can be controlled by modulating the dopamine concentration, pH and polymerization time [42]. Herein, the MAX1-dopamine gel was initially obtained via a polymerization time of three days. This dopamine polymerization time was chosen based on the literature showing that at pH 7.4 a maximal thickness of polydopamine film was obtained after 2–3 days of reaction [49]. We were intrigued to examine how at pH 7.4, in the presence of peptide gelator, factors such as dopamine polymerization time or dopamine concentrations could be used to further modulate the mechanical properties of the gel scaffold.

First, we examined the influence of polymerization time on the rheological properties of the gels. Although ~3 days was reported to be optimum for polydopamine film deposition on its own, this may not necessarily be the case in the presence of the peptide network. Extended reaction times might provide more extensive polydopamine network formation and the production of more quinone functionalities available for lysine crosslinking. Conversely, less reaction time should limit these processes. The MAX1-dopamine gel system was prepared as before but was studied 1, 2, 4 or 5 days following the initiation of peptide gelation. Rheological data at the different time points was compared to the rheological data collected 3 days (Figure 2D) post triggering assembly. Since peptide assembly and gelation occur promptly after the addition of buffer, changes to the mechanical rigidity of the gels will be mainly determined by the polydopamine formed within the examined time frame. At the examined time points, the initial recorded G′ values of the hybrid gel system were 8790 ± 1261, 12,863 ± 1059, 13,763 ± 705 and 14,018 ± 1458 Pa, 1, 2, 4, and 5 days post triggering the assembly, respectively. These G′ values are much higher than the G′ values displayed by MAX1 alone at the same time points (Figure S5), and in comparison to the highest G′ value displayed by MAX1 of about 300 Pa, represent a 29, 43, 46 and 47 fold increase in the mechanical rigidity of the gels at day 1, 2, 4 and 5, respectively. The data also verify that the polydopamine reaction is complete after about 3 days, since the G′ values level off after this time.

We next investigated the shear-thin/recovery properties of the MAX1-dopamine gels as a function of polymerization time. MAX1-dopamine gels that were allowed to polymerize only for 1 or 2 days before they were shear-thinned, displayed very low recovered G′ values of 701 ± 201 and 1049 ± 759 Pa, representing only 8 ± 2 and 8 ± 6% recovery, respectively. In comparison to the MAX1-dopamine gel that was shear-thinned 3 days post gelation (recovered G′ values of 12,020 ± 4638 Pa, 71 ± 25% recovery), the hybrid gels that were investigated following extended assembly time of 4 or 5 days, did not displayed a significant difference in the recovery behavior, showing recovered G′ values of 6212 ± 3598 and 9249 ± 1570 Pa, corresponding to 46 ± 28 and 66 ± 13% recovery, respectively. Again, the fact that dopamine gels significantly recover only after 3 days of reaction time, suggests that prior to this time point auto-polymerization of dopamine is yet to be complete.

TEM was performed on the MAX1-dopamine gels that were allowed to form for 2 and 4 days post triggering peptide assembly (Figures S6–S8). The local morphology of the fibrils isolated from MAX1 or MAX1-dopamine at day 2 and day 4 resembled the morphology observed 3 days after gelation was triggered (Figure 4). However, fibrils isolated from the MAX1-dopamine gel at 2 days incubation were slightly smaller in width than fibrils isolated from MAX1-dopamine gels, 3 or 4 days post triggering peptide assembly with dopamine, presumably due to a decrease in polydopamine formation.

**Figure 5:**
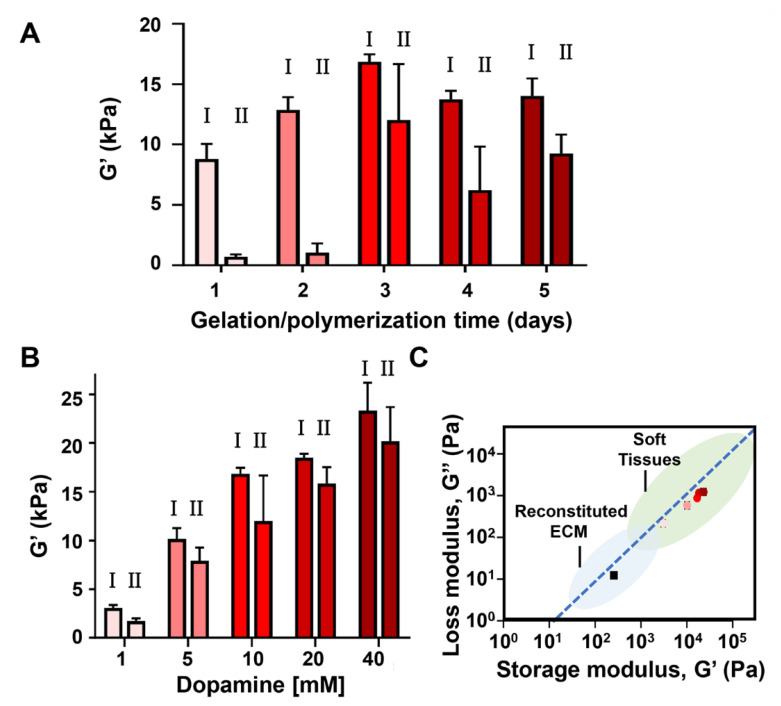
Rheological studies of the 1 wt.% MAX1-dopamine gel system. (**A**) G′ (0.2% strain, 6 rad/s) of pre-formed 1 wt.% MAX1 peptide gel assembled in the presence of 10 mM dopamine at 1, 2, 3, 4 or 5 days post triggering assembly. Left bar (I) represents the G′ value of the gel prior to shear-thinning at high strain (1000% strain, 30 s) and right bar (II) represents the recovered G′ value obtained 10 min after the gel was shear-thinned. (**B**) Rheological studies on pre-formed 1 wt.% MAX1 gel 3 days post triggering gelation in the presence of different dopamine concentrations. (**C**) Pre-sheared G′ and corresponding G″ values of pre-formed 1 wt.% MAX1-dopamine gel system with varying concentrations of dopamine (1, 5, 10, 20 and 40 mM), plotted in relation to G′/G″ value range of other viscoelastic materials reported in the literature [10]. The G′/G″ values of MAX1-dopamine are plotted as symbols in red shades corresponding to dopamine concentration presented in panel B. Black squares represent G′ and G″ of 1 wt.% MAX1 following 3-day assembly without dopamine. The blue dotted line indicates a G″ that is 10% of the G′, for reference. Full statistical analysis for panels A and B is given in the supporting info, Table S1.

With respect to incubation time, 3 days appears to be optimal. It is the shortest time leading to the highest recorded G′ values and affords gels with good recovery properties. Therefore, to investigate the influence of dopamine concentration on the mechanical rigidity of the MAX1-dopamine gel, rheological studies were conducted on gels 3 days after gelation was initiated in the presence of varied concentrations of dopamine (1, 5, 20 or 40 mM). Initial G′ values increased in correlation with dopamine concentrations. Relative to the G′ value for MAX1 gel (257 ± 20 Pa), G′ values of 3072 ± 292, 10,171 ± 1071, 18,503 ± 398 and 23,338 ± 2875 Pa were recorded in the presence of 1, 5, 20 or 40 mM dopamine, respectively, indicating a 12, 40, 72 or 91 fold increase in the mechanical rigidity. Shear-thin recovery studies revealed an improved recovery behavior of gels prepared with 20 or 40 mM of dopamine, corresponding to about 86% recovery (recovered G′ values of 15,836 ± 1694 and 20,171 ± 3540 Pa, respectively). TEM analysis was also performed on fibrils isolated from MAX1-dopamine gels prepared with either 1 or 40 mM of dopamine. In the presence of 40 mM dopamine (Figure S9), the isolated fibrils from the gel displayed similar local morphology and width as fibrils isolated from gels prepared with 10 mM dopamine. Interestingly, TEM micrographs of MAX1 prepared with 1 mM dopamine reveal the existence of two local morphologies (Figure S10), one population of long fibrils, similar to the ones that constitute the MAX1 gel (Figure 4A) and another population of short fibrils, that are comparable to fibrils from the MAX1-dopamine gel system presented earlier in this study (1 wt.% gel, 10 mM dopamine). These observations suggest that in the presence of low dopamine concentrations (1 mM), only minor deposition of polydopamine onto the fibrils occurs. G′ and G″ values of the gels were also plotted in relation to each other, as seen in Figure 5C. Previous rheological studies performed on reconstituted ECM materials such as fibrin and type I-collagen reported typical G′ and G″ values in the range of 10^1^–10^3^ and about 10^0^–10^2^ Pa, respectively, whereas some soft tissues display higher G′ and G″ values of about 10^3^–10^6^ and 10^2^–10^5^, respectively. In the absence of dopamine, MAX1 displays G′ and G″ values that make it suitable to serve as reconstituted ECM. The increase in G′ and G″ values of the hybrid MAX1-dopamine gel system expand its use to better mimic soft tissues, which can be useful for tailored applications.

**Figure 6:**
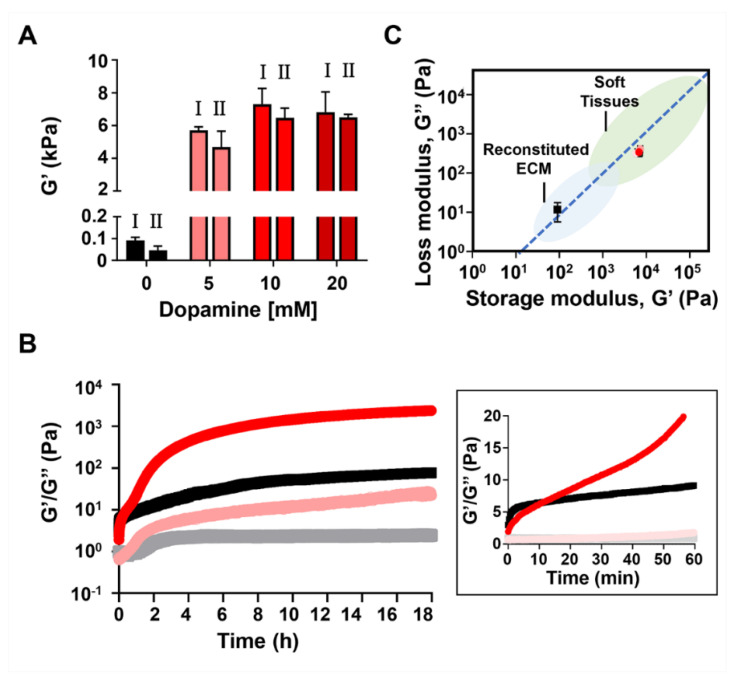
Rheological studies of the 0.5 wt.% MAX1-dopamine gel system. (**A**) Rheological studies on pre-formed 0.5 wt.% MAX1 gel 3 days post triggering gelation in the presence of different dopamine concentrations. Left bar (I) represents the G′ value of the gel prior to shear-thinning at high strain (1000% strain, 30 s) and right bar (II) represents the recovered G′ value obtained 10 min after the gel was shear-thinned. (**B**) Dynamic time sweep measurements of 0.5 wt.% MAX1 in the absence (black) and presence of 5 mM dopamine (red) monitoring the evolution of G′ and G″ (dark and light colors, respectively) as a function of time at 37 °C, pH 7.4. The insert depicts the first hour following the initiation of assembly, where the G′ and G″ values are plotted in a linear scale. (**C**) Pre-sheared G′ and corresponding G″ values of pre-formed 0.5 wt.% MAX1-dopamine gel system with varying concentrations of dopamine (5, 10 and 20 mM), plotted in relation to G′/G″ value range of other viscoelastic materials reported in the literature [10]. The G′/G″ values of MAX1-dopamine are plotted as symbols in red shades corresponding to dopamine concentration presented in panel A. Black squares represent G′ and G″ of 0.5 wt.% MAX1 following 3-day assembly without dopamine. The blue dotted line indicates a G″ that is 10% of the G′, for reference. Full statistical analysis for panel A is given in the supporting info, Table S1.

Next, we wanted to explore the ability of dopamine auto-polymerization to increase the mechanical rigidity of peptide-gels that are weaker than those formed by 1 wt.% MAX1. For that, we initiated the assembly of 0.5 wt.% MAX1 gel in the presence of varying concentrations of dopamine (5, 10 and 20 mM). In comparison to the previously examined 1 wt.% MAX1, at 0.5 wt.% MAX1, peptide assembly is slower, and a much less rigid gel is obtained, Figure 6A. In contrast to 1 wt.% MAX1, following shear-thinning the 0.5 wt.% gel only recovers to about 51% of its initial rigidity. It is worth mentioning that even at these low values of G′, 0.5 wt.% MAX1 is still a self-supporting gel. In the presence of 5, 10 and 20 mM dopamine, the G′ values were 5707 ± 215, 7304 ± 959 and 6809 ± 1241 Pa, respectively, which are about 63, 80 and 75 fold higher than the G′ values of 0.5 wt.% MAX1 alone (91 ± 14 Pa). Interestingly, upon shearing, all the hybrid gels showed more than 80% recovery, with recovered G′ values of 4687 ± 962, 6466 ± 593 and 6497 ± 192 Pa.

Using 0.5 wt.% MAX1, the kinetics of material formation were monitored directly in the rheometer for the MAX1-dopamine system. Pure 0.5 wt.% MAX1 was used as a control. As seen in Figure 6B, the gelation kinetics of MAX1 on its own is rapid and even at 0.5 wt.%, MAX1 forms gel within minutes. After the first 15 min following triggering gelation, similar G′ values were recorded for both MAX1 and the hybrid gel system. Yet, after this time point, in the presence of dopamine, the G′ values of the hybrid gel started to increase at a higher rate in comparison to the G′ values of MAX1 alone. Moreover, in the presence of dopamine we can see that the G′ values drastically increase after 40 min. The change in the kinetics profile after 40 min might suggest that at this time point a sufficient amount of polydopamine has formed in the system to effectively begin to crosslink the peptide gel. Again, plotting the G′ and G″ values of the hybrid gels formed with 0.5 wt.% MAX1 demonstrates the ability of dopamine to alter the gel’s viscoelastic properties to resemble values of soft biological tissues (Figure 6C).

## 3. Materials and Methods

### 3.1. Materials

Rink amide ChemMatrix^®^ resin, Oxyma, and all other Fmoc-protected amino acids were purchased from Novabiochem^®^, Sigma-Aldrich (St. Louis, MO, USA). 2-(6-Chloro-1*H*-benzotriazole1-yl)-1,1,3,3-tetramethylaminium hexafluorophosphate (HCTU) was purchased from Chem Impex International (Wood Dale, IL, USA). Piperidine and ethanedithiol (98+%) were purchased from Alfa Aesar (Tewksbury, MA, USA). Trifluoroacetic acid (TFA, 97%), anisole (99%), thioanisole (≥99%), 4-(2-hydroxyethyl)-1-piperazineethanesulfonic acid (HEPES) and dopamine hydrochloride (H8502, ≥98%) were purchased from Sigma Aldrich (St. Louis, MO, USA). *N,N′*-Diisopropylcarbodiimide (DIC, 99%), *N,N*-Diisopropylethylamine (DIEA), Dimethylformamide (DMF, 99.9%), Dichloromethane (DCM, ≥99.8%), Diethyl ether and acetonitrile were purchased from Fisher Scientific (Fisher Scientific, Fairlawn, NJ, USA).

### 3.2. Methods

#### 3.2.1. Peptide Synthesis

MAX1 peptide was synthesized by a standard Fmoc-solid phase peptide synthesis, using a Liberty Blue™ automated microwave peptide synthesizer (CEM) with H-Rink amide ChemMatrix^®^ resin. Resin-bound peptide was cleaved and side chain-deprotected using a cleavage cocktail of TFA:thioanisole:ethanedithiol:anisole (90:5:3:2) for 3 h under argon. Crude peptide was purified by RP-HPLC using a preparative Vydac C18 peptide column at 40 °C. Gradients of standard A (0.1% TFA in water) and standard B (0.1% TFA in 9:1 acetonitrile/water) were used as follows: an isocratic gradient from 0 to 2 min at 0% standard B, a linear gradient from 0 to 15% standard B for 8 min and a linear gradient of 15 to 100% standard B over an additional 149 min. The peptide eluted at approximately 36 min, lyophilized and then analyzed using analytical HPLC and LC-MS. Analytical HPLC chromatograms and ESI (+) mass spectra of the pure peptide are provided in Figure S11.

#### 3.2.2. Gel Preparation

MAX1 gels were prepared by dissolving lyophilized peptide in water to obtain 2× concentrated (wt.%) peptide stock solution. Peptide assembly and gelation was initiated by mixing together equal volumes of the 2× peptide stock solution and chilled 2× HEPES buffer solution (150 mM HEPES, 300 mM NaCl, pH 7.4) on ice. The final mixed solution (1× wt.% peptide in 75 mM HEPES, 150 mM NaCl, pH 7.4) was then incubated for the desired amount of time at 37 °C, resulting with a self-supporting gel. Preparation of MAX1-dopamine gels was done as follows: dopamine was dissolved in water to obtain 100× concentrated [M] dopamine solution. The dopamine solution was added to the peptide stock solution in a volume that accounts for 2% of the total 2× peptide solution and mixed. Peptide assembly and gelation was initiated by mixing equal volumes of the 2× peptide/dopamine stock solution with chilled 2× HEPES buffer solution (150 mM HEPES, 300 mM NaCl, pH 7.4) on ice, followed by incubation at 37 °C. For example, to prepare 200 µL of 1 wt.% (~3 mM) MAX1 gel with 10 mM dopamine, 98 µL of 2 wt.% MAX1 (6.14 mM) were mixed with 2 µL of 1 M dopamine in water. The resulting 100 µL mixture of MAX1-dopamine (~6 mM MAX1, 20 mM dopamine) was mixed with 100 µL 2× HEPES buffer solution (150 mM HEPES, 300 mM NaCl, pH 7.4) and transferred to 37 °C.

#### 3.2.3. Rheological Studies

Rheological measurements were performed on pre-formed gels using an AR G2 rheometer (TA Instruments) equipped with an 8 mm stainless steel parallel plate geometry tool. 100 µL of MAX1 or MAX1/dopamine gels in HEPES buffer (0.5 or 1 wt.% gels) were prepared as described above in Corning^®^ Costar^®^ Transwell^®^ cell culture inserts positioned in a 24-well plate (Corning™ 3422, Millipore Sigma, St. Louis, MO, USA). Following incubation at 37 °C for the desired amount of time (1, 2, 3, 4- or 5-days post triggering the assembly), gels were removed from the inserts and placed on a heated rheometer stage (37 °C). The geometry was lowered onto the sample to a gap height of 0.5 mm and standard S6 oil was placed around the geometry tool to prevent evaporation during the measurements. Rheological studies of the gels included a dynamic frequency sweep (frequency range of 0.1 to 100 rad/s) collected at 0.2% strain and a dynamic strain sweep (0.1 to 1000 strain%) collected at a constant frequency of 6 rad/s. Shear-thinning/recovery cycles of the gels were performed as follows: 10 min time-sweep at the LVE regime (0.2% strain, 6 rad/s), then shearing for 30 s under high strain (1000% strain, 6 rad/s), following by a 10 min time-sweep at the LVE regime (0.2% strain, 6 rad/s). For dynamic time sweeps measurements of gels formed on the rheometer stage, a 25 mm stainless steel parallel plate geometry was used. The 0.5 wt.% gels were prepared as described above and quickly transferred (300 µL) to the rheometer stage, which was pre-equilibrated at 5 °C. The temperature was ramped linearly to 37 °C to initiate assembly (90 s) and the G′ and G″ were monitored as a function of time for 18 h (0.2% strain, 6 rad/s). Rheological data represent the average G′ and G″ obtained from three independent measurements.

#### 3.2.4. Circular Dichroism (CD) Measurements

CD spectra were collected with a Jasco J-1500 circular dichroism spectrometer, using a 0.1 mm pathlength quartz cuvette. Data acquisition was performed over a wavelength range of 200–260 nm in steps of 1 nm with an average time of 2 s. Each spectrum represents an average of three spectra collected subsequently. Dynode values were monitored and kept under 700 units for all measurements. A background spectrum of blank buffer or water was subtracted from the sample spectra and the mean residue ellipticity [θ] was calculated from the equation: [θ] = θobs/(10 × l × c × r) where θobs is the observed ellipticity (millidegrees), l is the length of the cell (0.01 cm), c is the molar concentration of the peptide (M), and r is the number of residues (20). The exact peptide stock concentrations were determined by the UV-absorbance of a diluted aqueous peptide solution of MAX1 at 220 nm (Agilent 8453 UV-Visible Spectroscopy System, Agilent Technologies, Wilmington, DE, USA), according to Beer–Lambert law, using a molar extinction coefficient at 220 nm of 15,750 cm^−1^ M^−1^. Temperature-dependent CD spectra were collected from 2 °C to 92 °C in an ascending 5 °C stepped ramp. The sample was allowed to equilibrate for 10 min at every temperature point before measurement.

#### 3.2.5. Transmission Electron Microscopy

1 wt.% MAX1 gel or 1 wt.% MAX1 gel with 10 mM dopamine were prepared in Eppendorf tubes as described above and incubated at 37 °C for three days. To allow visualization of distinct fibers, gel samples were diluted 50× into water. A 5 µL drop of the diluted peptide solution was placed on a 200 mesh copper grid covered by carbon film (Electron Microscopy Science, Hatfield, PA, USA) for 1 min and blotted by filter paper. Subsequently, for washing, 5 µL of water were added to the grid for several s and blotted by filter paper. Immediately after that, 0.75% uranyl formate was added to the grid and allowed to stand for 1 min, then blotted with a filter paper and left to air dry. Images were taken with a Technai T12 (FEI Company, Hillsboro, OR, USA) at 80 kv accelerating voltage. Average fibril width was measured via ImageJ software (National Institutes of Health, Bethesda, MD, USA) by taking 80 (MAX1) or 193 (MAX1/dopamine) independent measurements from distinct fibrils in the field of view of fibrils observed at 3 separate micrographs, representing different location of the fibrils.

## 4. Conclusions

Dopamine self-polymerization can be used as a simple, yet powerful approach to modulate the viscoelastic properties of peptide-based gels. Rheological studies showed that the storage modulus of peptide gels can be increased up to 91-fold when peptide gelation is triggered in the presence of dopamine, while retaining the gel’s shear thin-recovery behavior. Moreover, at the low peptide concentration of 0.5 wt.% MAX1, not only does polydopamine increase gel rigidity, but it also further improved the recovery behavior of the gel upon shear-thinning. In this study we examined how factors such as dopamine self-polymerization time, peptide and dopamine concentration can alter the viscoelastic properties of the resultant gels, when prepared at pH 7.4, 37 °C. We observed that an optimal incubation time of 3 days is needed to afford gels with good recovery behavior upon shear-thinning. Peptide gelation in the presence of dopamine concentrations ≥5 mM leads to an increase in the mechanical rigidity of both 0.5 and 1 wt.% gels. The highest increase in rigidity was observed for 1 wt.% peptide gel prepared in the presence of 40 mM dopamine, the maximal concentration investigated herein. Other factors that might affect the kinetics of peptide self-assembly and/or dopamine self-polymerization processes, such as solution pH and gelation temperature, can be further adjusted to modulate and fine-tune the viscoelastic properties of the hybrid gels for tailored applications. The hybrid peptide–polydopamine gel matrix offers not only a peptide-gel scaffold with improved mechanical properties but may also benefit from the properties of the polydopamine in future designs. Collectively, such hybrid gels might find use in advanced applications such as 3D bioprinting.

## Data Availability

Not applicable.

## Supplementary Materials

The following are available online, Figure S1. Temperature-dependent CD spectra of 1 wt.% MAX1 in water. Figure S2. θ_216_ values of 10 mM dopamine in HEPES buffer, 37 °C. Figure S3. Temperature-dependent CD spectra of 10 mM dopamine in HEPES buffer. Figure S4. TEM micrographs 1 wt.% MAX1 fibrillar gel following 3 days polymerization with 10 mM dopamine. Figure S5. Rheological studies of 1 wt.% MAX1 gels as a function of time. Figure S6. TEM micrographs 1 wt.% MAX1 fibrillar gel following 2- or 4-day polymerization with 10 mM dopamine. Figure S7. TEM micrographs 1 wt.% MAX1 fibrillar gel following 2 days polymerization with of 10 mM dopamine. Figure S8. TEM micrographs 1 wt.% MAX1 fibrillar gel following 4 days polymerization with of 10 mM dopamine. Figure S9. TEM micrographs 1 wt.% MAX1 fibrillar gel following 3 days polymerization with of 40 mM dopamine. Figure S10. TEM micrographs 1 wt.% MAX1 fibrillar gel following 3 days polymerization with of 1 mM dopamine. Figure S11. Analytical HPLC and ESI (+) mass spectrum of purified MAX1 peptide. Table S1. Statistical analysis for rheological measurements.
