# Supplementary material for: Dopamine Self-Polymerization as a Simple and Powerful Tool to Modulate the Viscoelastic Mechanical Properties of Peptide-Based Gels
Source: Molecules. 2021 Mar 4;26(5):1363. doi: 10.3390/molecules26051363 (PMC7961423; doi:10.3390/molecules26051363)
Supplement: Supplementary file 1 [file molecules-26-01363-s001.pdf]

Supporting info

Dopamine Self-Polymerization as a Simple and Powerful Tool to Modulate the Viscoelastic Mechanical Properties of Peptide-Based Gels

Galit Fichman [1] and Joel P. Schneider [1,*]

[1] Chemical Biology Laboratory, National Cancer Institute, National Institutes of Health, Frederick, Maryland, USA; gali.fichman@nih.gov (G.F); Joel.Schneider@nih.gov (J.P.S)

* Correspondence: Joel.Schneider@nih.gov

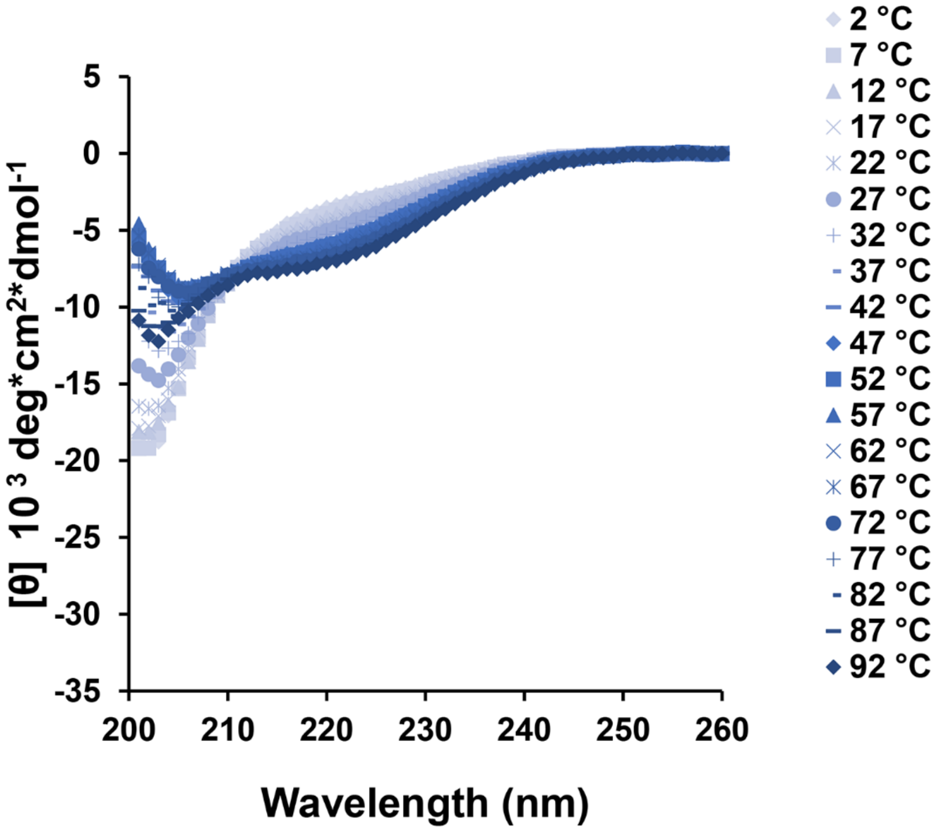

Figure S1. Temperature-dependent CD spectra of 1 wt% MAX1 in water.

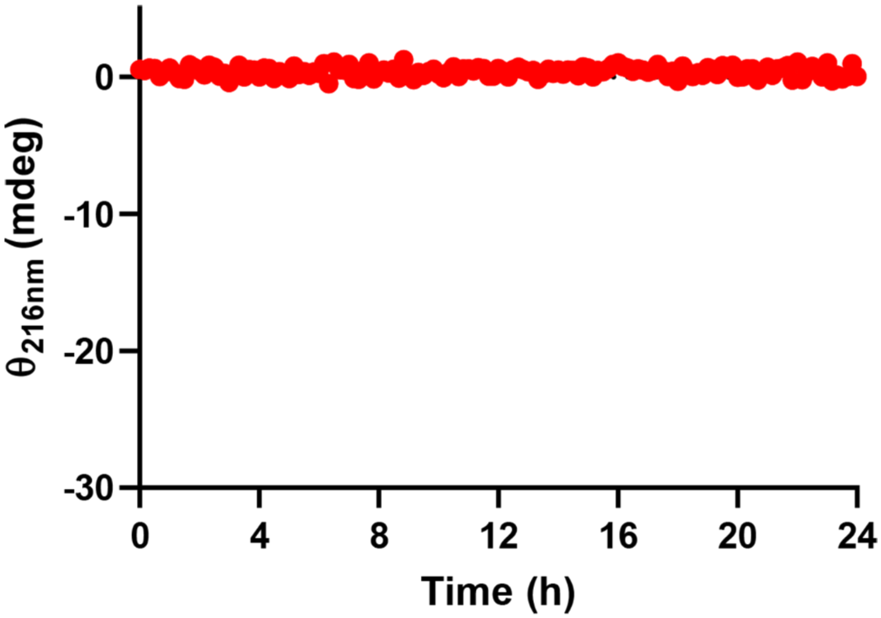

Figure S2. θ_{216} values of 10 mM dopamine in HEPES buffer (75 mM HEPES, 150 mM NaCl, pH 7.4) collected at 37°C as a function of time.

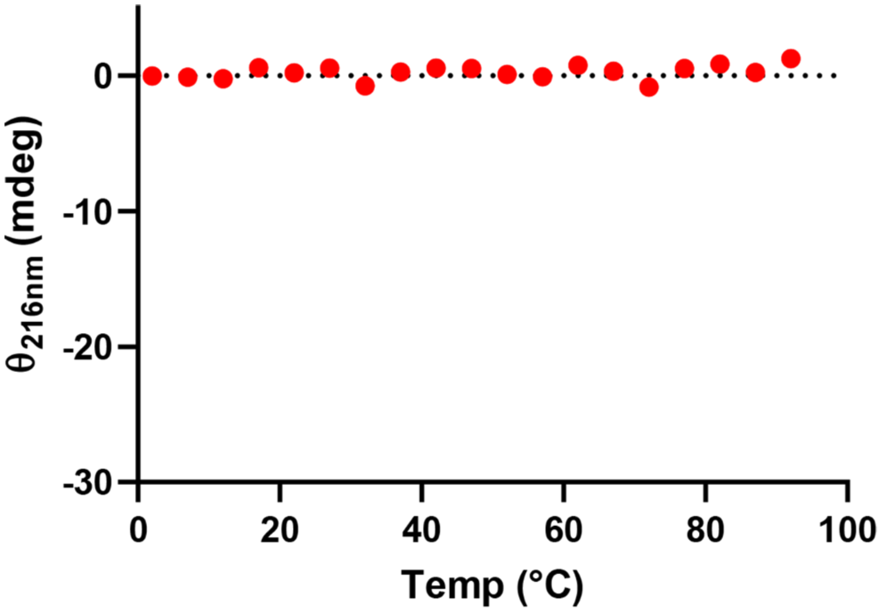

Figure S3. Temperature-dependent CD spectra of 10 mM dopamine in HEPES buffer (75mM HEPES, 150 mM NaCl, pH 7.4).

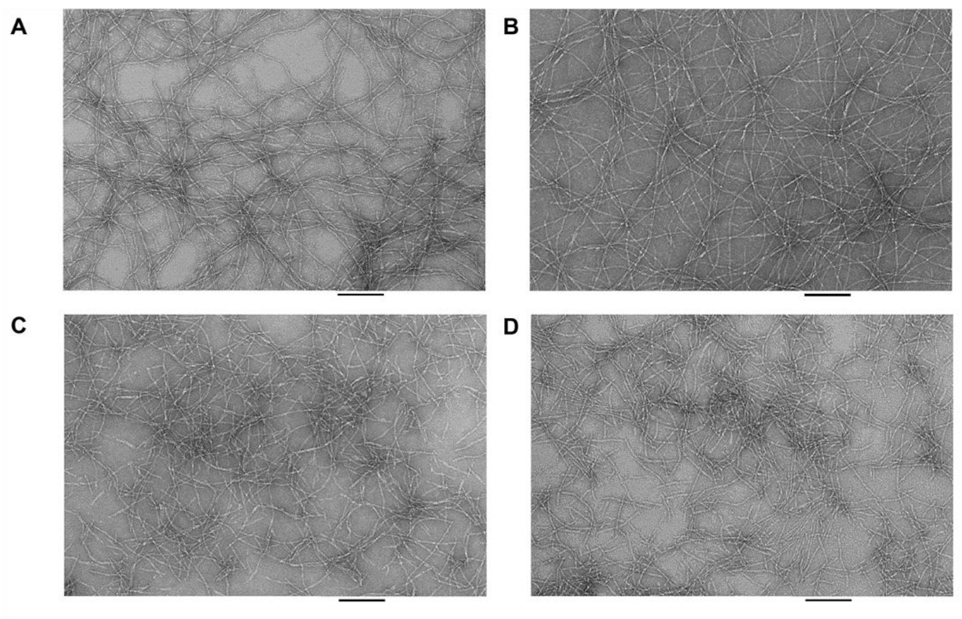

Figure S4. TEM micrographs showing fibrils isolated from 1 wt% MAX1 fibrillar gel networks 3 days after gelation is triggered in the absence or presence of 10 mM dopamine (A, B and C, D respectively). Scale bar =100 nm.

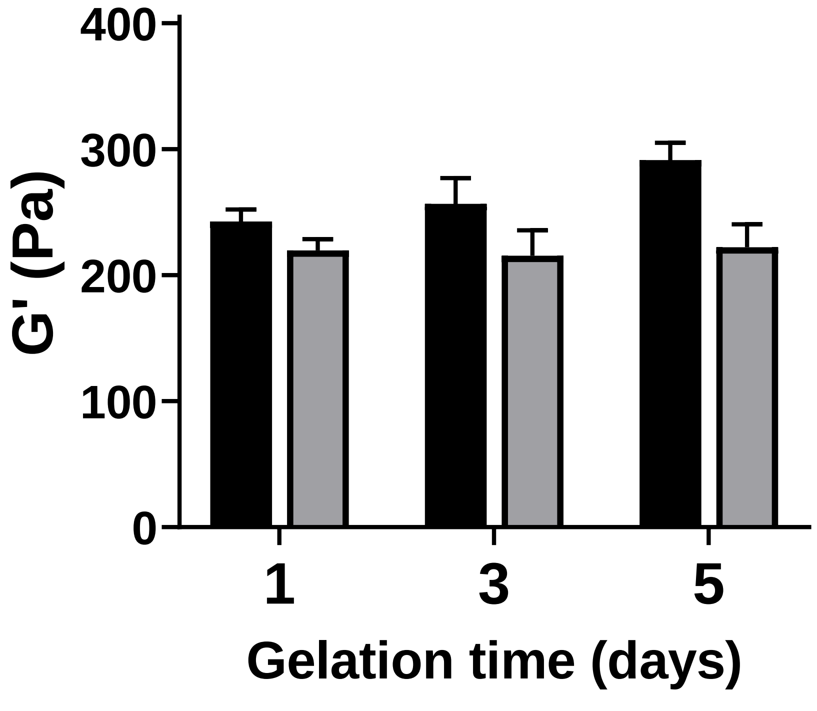

Figure S5. Rheological studies of MAX1 gels as a function of time. G’ (0.2% strain, 6 rad/sec) of pre-formed 1wt% MAX1 peptide gel at 1, 3 or 5 days post triggering assembly. Left bar (black) represents the recorded G’ value of the gel prior to shear-thinning at high strain (1000% strain, 30 sec) and right bar (gray) represents the recovered G’ value obtained 10 minutes after the gel was shear-thinned.

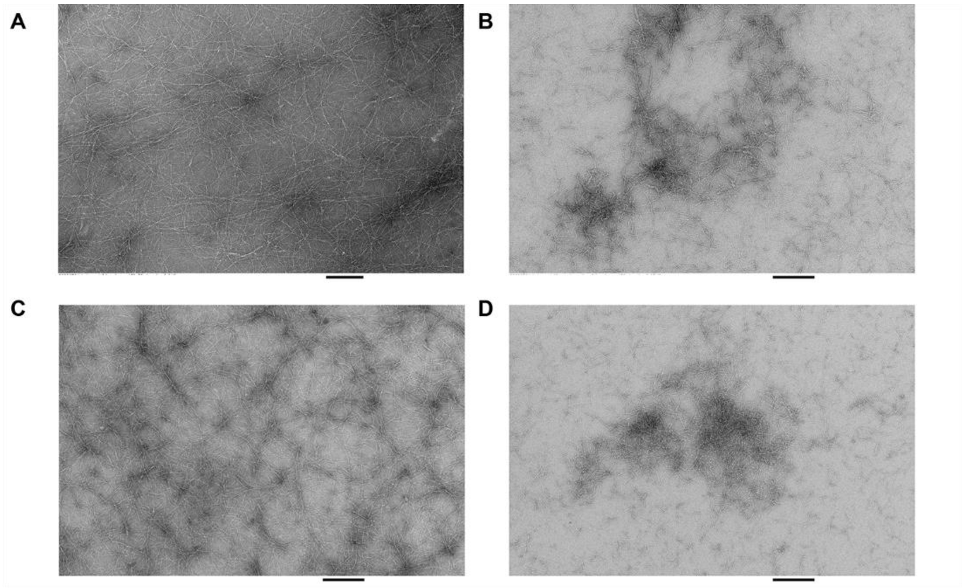

Figure S6. TEM micrographs showing fibrils isolated from 1 wt% MAX1 fibrillar gel networks 2 (A,B) or 4 (C, D) days after gelation is triggered in the absence (A, C) or presence (B,D) of 10 mM dopamine. Scale bar =200 nm.

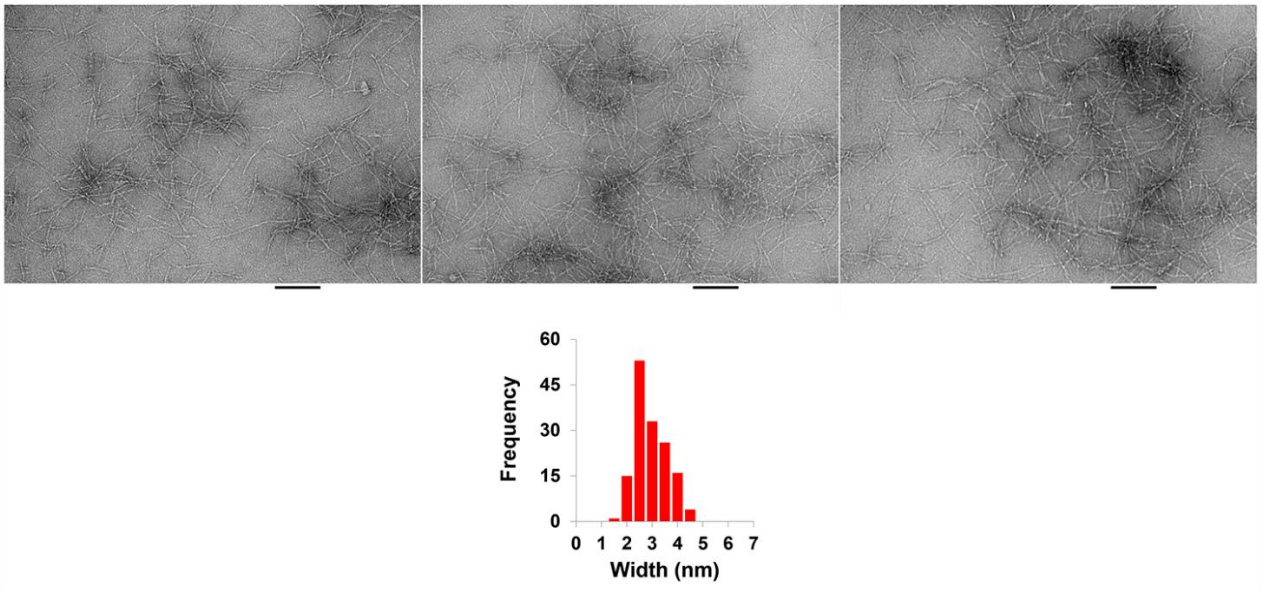

Figure S7. TEM micrographs showing fibrils isolated from 1 wt% MAX1 fibrillar gel networks 2 days after gelation is triggered in the presence of 10 mM dopamine. Scale bar =100 nm. Widths of individual fibrils of each sample were determined using ImageJ software, by measuring width of fibrils from 3 separate micrographs, representing different location of the fibrils on the grid, n=148.

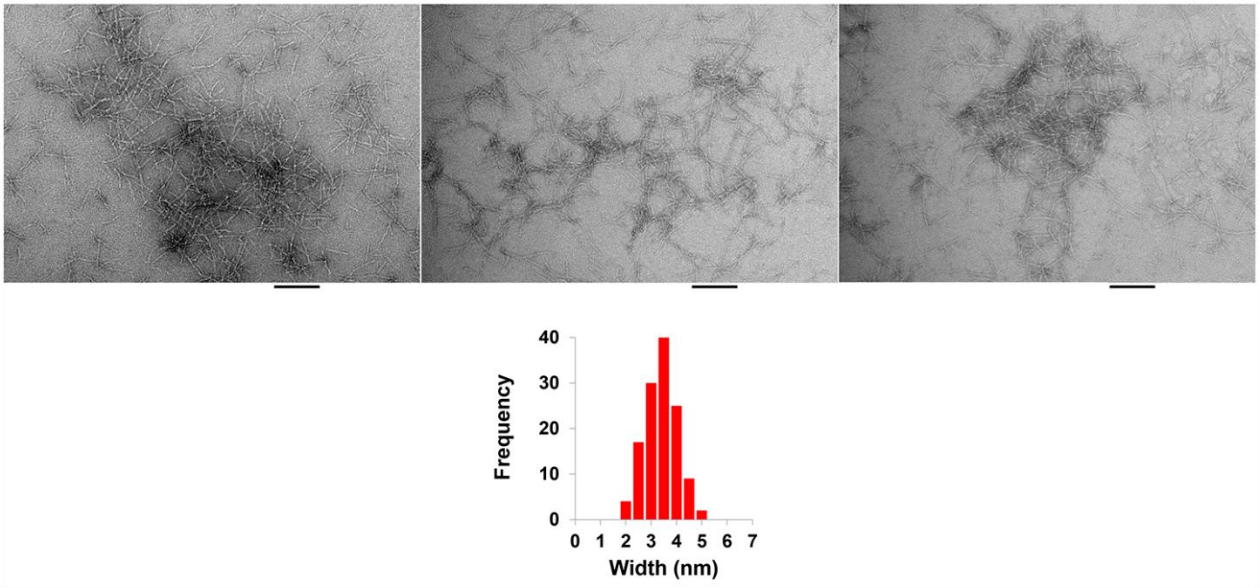

Figure S8. TEM micrographs showing fibrils isolated from 1 wt% MAX1 fibrillar gel networks 4 days after gelation is triggered in the presence of 10 mM dopamine. Scale bar =100 nm. Widths of individual fibrils of each sample were determined using ImageJ software, by measuring width of fibrils from 3 separate micrographs, representing different location of the fibrils on the grid, n=127.

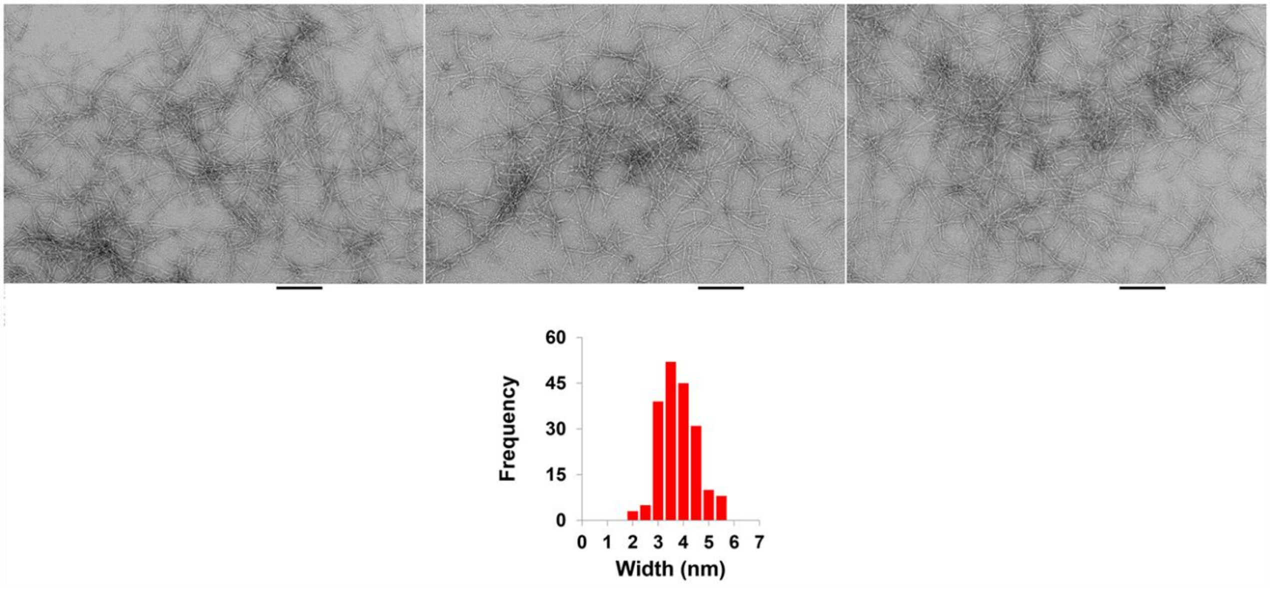

Figure S9. TEM micrographs showing fibrils isolated from 1 wt% MAX1 fibrillar gel networks 3 days after gelation is triggered in the presence of 40 mM dopamine. Scale bar =100 nm. Widths of individual fibrils of each sample were determined using ImageJ software, by measuring width of fibrils from 3 separate micrographs, representing different location of the fibrils on the grid, n=193.

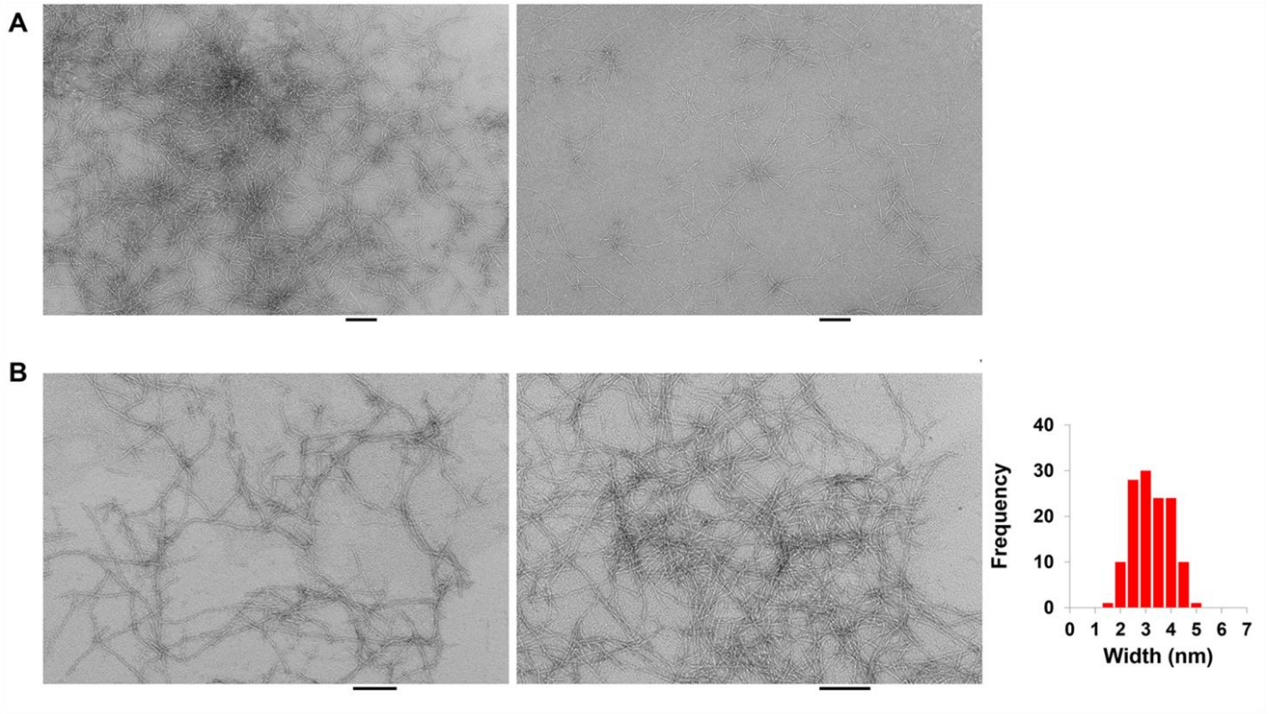

Figure S10. TEM micrographs showing fibrils isolated from 1 wt% MAX1 fibrillar gel networks 3 days after gelation is triggered in the presence of 1 mM dopamine. Scale bar =100 nm. **A.** Micrographs at lower magnifications showing the existence of two populations of long and short fibrils (left and right panel, respectively). **B.** Widths of individual fibrils of each sample were determined using ImageJ software, by measuring width of fibrils from 2 separate micrographs, representing different location of the fibrils on the grid, n=128.

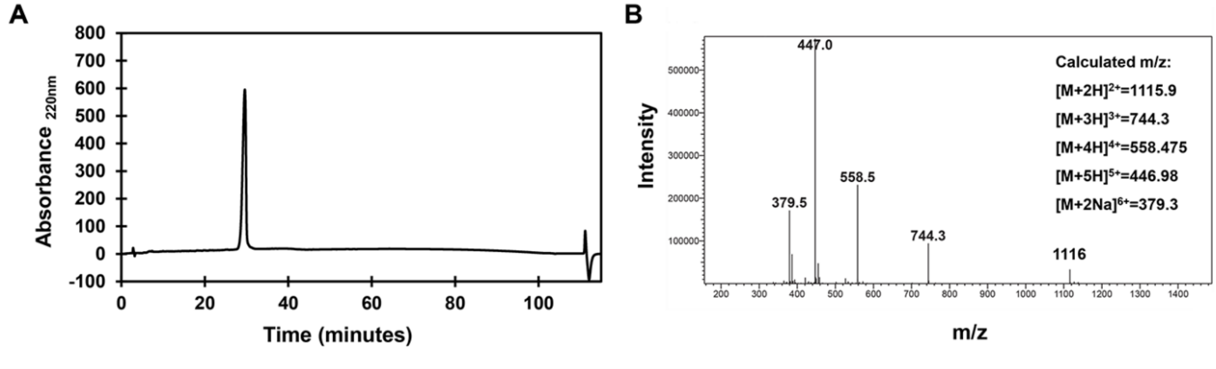

Figure S11. (**A**) Analytical HPLC (0%-100% B over 100 min) and (**B**) ESI (+) mass spectrum of purified MAX1 peptide.

Figure	Groups compared	Statistical significance
Figure 5A Gelation/polymerization time	1 day (I) vs 2 days (I)	*
	2 days(I) vs 3 days(I)	**
	3 days (I) vs 4 days (I)	**
	4 days (I) vs 5 days (I)	ns
Figure 5A Gelation/polymerization time	1 day (II) vs 2 days (II)	ns
	2 days(II) vs 3 days(II)	*
	3 days (II) vs 4 days (II)	ns
	4 days (II) vs 5 days (II)	ns
Figure 5A Gelation/polymerization time	1 day (I) vs (II)	***
	2 days(I) vs (II)	****
	3 days(I) vs (II)	ns
	4 days(I) vs (II)	*
	5 days(I) vs (II)	*
Figure 5B Dopamine [mM]	1 mM (I) vs 5 mM (I)	***
	5 mM (I) vs 10 mM (I)	***
	10 mM (I) vs 20 mM (I)	*
	20 mM (I) vs 40 mM (I)	*
Figure 5B Dopamine [mM]	1 mM (II) vs 5 mM (II)	**
	5 mM (II) vs 10 mM (II)	ns
	10 mM (II) vs 20 mM (II)	ns
	20 mM (II) vs 40 mM (II)	ns
Figure 5B Dopamine [mM]	1 mM (I) vs (II)	**
	5 mM (I) vs (II)	ns
	10 mM (I) vs (II)	ns
	20 mM (I) vs (II)	ns
	40 mM (I) vs (II)	ns
Figure 6A Dopamine [mM]	0 mM (I) vs 5 mM (I)	****
	5 mM (I) vs 10 mM (I)	*
	10 mM (I) vs 20 mM (I)	ns
Figure 6A Dopamine [mM]	0 mM (II) vs 5 mM (II)	**
	5 mM (II) vs 10 mM (II)	ns
	10 mM (II) vs 20 mM (II)	ns
Figure 6A Dopamine [mM]	5 mM (I) vs 5 mM (II)	ns
	10 mM (I) vs 10 mM (II)	ns
	20 mM (I) vs 20 mM (II)	ns

Table S1. Statistical analysis for rheological measurements. Statistical analysis was performed using GraphPad Prism 8.3.8 (GraphPad Software). Differences were calculated using an unpaired two-tailed Student's *t*-test with *P* values being shown. Significance differences of *, **, ***, **** represent P values of $P \leq 0.05$, $P \leq 0.01$, $P \leq 0.001$ and $P \leq 0.0001$. ns=no significance difference, $P > 0.05$.
